# The Four Epochs of Woman’s Life

**Published:** 1902-02

**Authors:** 


THE FOUR EPOCHS OF WOMAN'S LIFE. A Study in Hygiene. By ANNA M. GALBRAITH, M. D., Author of "Hygiene and Physical Culture for Women;" Fellow of the New York Academy of Medicine, etc. With an Introductory Note by John H. Musser, M. D., Professor of Clinical Medicine, University of Pennsylvania. Duodecimo volume of 200 pages. Philadelphia and London: W. B. Saunders & Co. 1901. [Price, cloth, $1.25 net.]

The present is preëminently an age of preventive medicine. In large ways by national, state, and municipal health regulations, in small ways by instruction, both public and private, health boards, medical societies and physicians and intelligent laymen are striving to limit the spread of disease, to preserve health rather than to cure disease. With this spirit as the motive power, Dr. Galbraith has written a book on sexual hygiene for women. The work is brief—scarce 200 pages are filled, but it presents clearly, concisely and modestly those facts of anatomy and physiology and that practical hygiene, knowledge of which will save a woman anxiety, suffering and actual disease. The book has much worthy of commendation. The chapter upon the menopause is one which might have been improved. The author here fails to make clear to the reader the necessity for early medical attention in cases where symptoms point to malignant disease of the uterus.

There is much in the volume which the husband as well as the wife should know.—M.J.F.

